# Effects of music intervention during caesarean delivery on anxiety and stress of the mother a controlled, randomised study

**DOI:** 10.1186/s12884-018-2069-6

**Published:** 2018-11-03

**Authors:** Philip Hepp, Carsten Hagenbeck, Julius Gilles, Oliver T. Wolf, Wolfram Goertz, Wolfgang Janni, Percy Balan, Markus Fleisch, Tanja Fehm, Nora K. Schaal

**Affiliations:** 1 Clinic for Gynecology and Obstetrics, Helios University Hospital Wuppertal, University Witten/Herdecke, Heusnerstr 40, 42283 Wuppertal, Germany; 2 Clinic for Gynecology and Obstetrics, Heinrich-Heine-University, Düsseldorf, Germany; 3 Department of Cognitive Psychology, Institute of Cognitive Neuroscience, Faculty of Psychology, Ruhr-University Bochum, Bochum, Germany; 4 Musikerambulanz, Heinrich-Heine-University, Düsseldorf, Germany; 5 Clinic for Gynecology and Obstetrics, University Hospital Ulm, Ulm, Germany; 6 Department of Experimental Psychology, Heinrich-Heine-University, Düsseldorf, Germany

**Keywords:** Caesarean, Anxiety, Stress, Music intervention

## Abstract

**Background:**

Stress and anxiety during pregnancy and childbirth have negative consequences for both mother and child. There are indications that music has a positive effect in this situation. The present study investigates the influence of music during the caesarean on anxiety and stress of the expectant mother.

**Methods:**

The SAMBA study is a single-centre, controlled, randomized study including 304 patients. Women in the intervention group heard music via loudspeakers from one of four self-selected genres. The control group had standard treatment without music. The caesarean was performed in regional Anesthesia. At admission, at skin incision, during skin suture and two hours after completion of surgery, different subjective (State-Trait Anxiety Inventory, visual analogue scale for anxiety) and objective parameters (salivary cortisol/amylase, heart rate, blood pressure) were collected. Mixed-factorial Analysis of variances as well as independent sample t-tests were applied for data analysis.

**Results:**

At skin suture, significantly lower anxiety levels were reported in the intervention group regarding State anxiety (31.56 vs. 34.41; *p* = .004) and visual analogue scale for anxiety (1.27 vs. 1.76; *p* = .018). Two hours after surgery, the measured visual analogue scale for anxiety score in the intervention group was still significantly lower (0.69 vs. 1.04; *p* = .018). The objective parameters showed significant differences between the groups in salivary cortisol increase from admission to skin suture (12.29 vs. 16.61 nmol/L; *p* = .043), as well as systolic blood pressure (130.11 vs. 136.19 mmHg; *p* = .002) and heart rate (88.40 vs. 92.57/min; *p* = .049) at skin incision.

**Conclusions:**

Music during caesarean is an easy implementable and effective way of reducing stress and anxiety of the expectant mother.

**Trial registration:**

German registry for clinical trials (DRKS00007840). Registered 16/06/2015. Retrospectively registered.

## Background

Almost one in three women delivers by caesarean in Germany. Thus it is the most common abdominal surgery and one of the most common operations. Although the circumstances in almost all cases give cause for joy, it is also a feared event, which is associated with a significant level of stress for the patient [1, 2]. Studies have shown adverse effects of maternal stress on the foetus and on psychological development later in life [3, 4]. In addition, it is known that increased levels of stress and anxiety can negatively affect pain perception and the usage of analgesics postoperatively [5, 6] as well as the new mothers lactation [7, 8]. In view of the limited pharmacological options of intervention for pregnant women, the need for alternative, low-risk approaches to positively influence anxiety and stress arises.

In this regard, the positive effect of music on anxiety and stress is one of the oldest treatment approaches. Even Asclepius attributed a healing power to music [9]. Its positive influence in various medical interventions across all disciplines has been repeatedly examined [10–13]. Supported by a recent review by Hole et al. in the Lancet, these results also drew the attention of the wider public [14].

Nevertheless, data for music during caesarean are sparse and inconclusive [15]. Two randomized studies examining the influence of music during the pre-operative waiting time could show a positive influence of music on non-validated questionnaires as well as heart rate and heart rate variability as a surrogate for stress and anxiety. In part, this effect could still be detected six hours after the caesarean [16, 17]. A Cochrane analysis [15] investigating the effect of music during the procedure could only identify one randomized study that was able to show a positive effect of music intervention on heart rate at the end of the caesarean in 64 participants [18]. Due to their study design, no further conclusions on subjective and objective perception of anxiety were possible. Another study examined the influence of music during caesarean delivery under general anesthesia on postoperative pain and could not show any effect [19].

Therefore, the aim of the present study is to systematically examine the anxiolytic and stress reducing effect of a music intervention during the caesarean on the wake patient using validated questionnaires and a comprehensive set of objective measurements (salivary cortisol/amylase, blood pressure and heart rate).

## Methods

The *SAMBA* **(S**ectio Caesarea und die **A**uswirkung von **M**usik**B**egleittherapie auf **A**nxiolyse; English: Caesarean and the effect of music intervention on anxiety) study is a single-centre, controlled, randomized trial conducted at the University Hospital Dusseldorf, Germany. The study adheres to the CONSORT guidelines.

### Ethics, consent and permissions

The study protocol was approved by the ethics committee of the Medical Department of the Heinrich-Heine-University in Dusseldorf (No.: 3625) in accordance with the declaration of Helsinki and registered in the German registry for clinical trials (DRKS00007840) and with the WHO (Universal Trial Number U1111–1173-3204). Upon reasonable request the study protocol can be obtained via email from the corresponding author. All eligible patients gave informed written consent prior to participation.

### Participants

From March 2015 to August 2017, pregnant women with an indication for primary caesarean in regional anesthesia and adequate German language comprehension were recruited.

We only included patients with normal hearing abilities. Additionally, the patients were only included when no serious comorbidities (according to the physician’s assessment) were present, no significantly increased surgical risk (e.g. placental disturbances) were identified preoperatively and only if no serious condition of the foetus was known. Furthermore, only patients without any generalized anxiety disorder or other serious mental alterations were included in the study.

### Outcomes, measuring instruments and procedure

The impact of music during caesarean delivery on anxiety and stress measured by State-Trait Anxiety Inventory (STAI), a visual analogue scale depicting anxiety (VAS-A) as well as on salivary cortisol and alpha-amylase were pre-specified as primary outcome measures.

To measure subjective anxiety, STAI and VAS-A were used. The STAI is an introspective inventory comprising 40 self-report items pertaining to anxiety [20]. It distinguishes between two questionnaires with 20 items each, one measuring anxiety perceived in the current situation (STAI-state) and the other evaluating a general tendency towards anxiety (STAI-trait). Participants are asked to give a response to each item on a 4-point Likert scale. A total value is calculated (possible range 20–80 for each questionnaire). Higher scores reflect higher levels of anxiety. The VAS-A comprises a 10 cm line, on which the participant marks her current degree of anxiety with the left end of the line being labelled “no anxiety” and the right end being labelled “maximum anxiety”. For analysis the marking is then measured in mm from the left end.

Saliva samples were collected in order to determine cortisol and alpha-amylase levels as objective measures of stress. Salivary cortisol is a marker of the activation of the hypothalamic-pituitary-adrenal axis, whereas salivary alpha-amylase is an indirect marker of the autonomic activity [21]. For the saliva samples, patients had to thoroughly insalivate a cotton swab. The saliva samples were kept frozen at − 18 degrees until analysed, following the methods described elsewhere [22]. Heart rate and blood pressure values were taken from the anaesthesia records.

After inclusion, the computer-assisted randomization took place and divided the patients into the music group vs. control group in the ratio of one to one. Women in the music group chose their preferred music genre from lounge, classical, jazz, and meditation music.

**Fig. 1:**
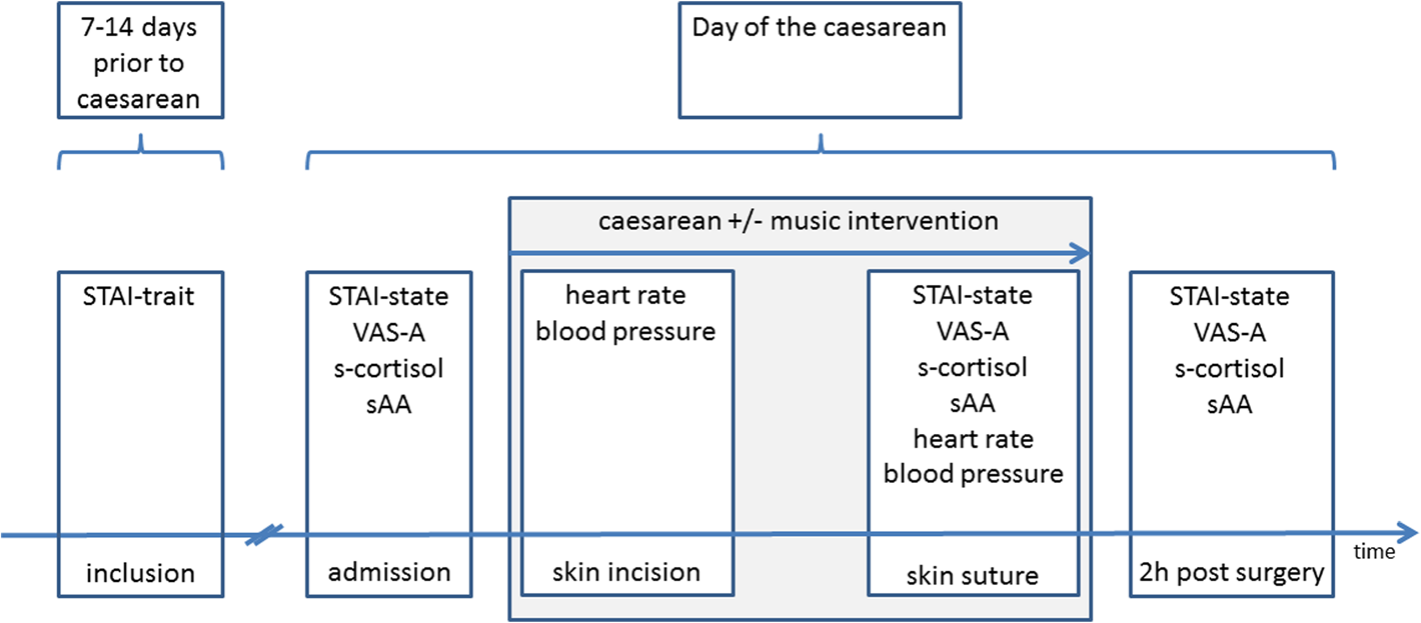
Flow chart of the study with details of the obtained data for each measurement time point

The participating women filled in the STAI-trait when they came to the routine surgical preparation appointment 7 to14 days before the caesarean. At admission on the day of the scheduled caesarean, the VAS-A, the STAI-state and the first saliva sample were taken during the routine cardiotocogram. There was no routine preoperative medication in either group. Intraoperative, the parameters blood pressure and heart rate were recorded at skin incisions and suture. During skin suture, a second saliva sample was obtained and the patient answered the STAI-state questionnaire and the VAS-A. At the end of post-operative monitoring two hours after skin suture, a third saliva sample was taken. Furthermore, the participant answered the STAI-state questionnaire and VAS-A for a third time. Additionally, questions were asked about the music experience. Figure 1 shows the study procedure with the measurement time points schematically.

### Intervention

For the music group, the music intervention started when the participant entered the operating theatre. The music was played on a CD player (TEAC CR-H 500 CD receiver) using a speaker system from Cambridge Audio 300. The participant was presented with music continuously in a standardized volume determined on the device and measured at the participants’ head of 55 dB (A). All music titles had a slow tempo of 60–80 bpm in common and followed the recommendations made by Nilsson [23]. The music pool contained 60 songs. 15 tracks were each assigned to one of the 4 different genres of music. The control group received no music.

### Statistical analysis

The statistical software package SPSS 24 (IBM Inc., Armonk, NY) was used for all data analyses. The group affiliation was coded and therefore the analysis was performed blind. In order to check for differences in environmental parameters between groups, which could influence the results, we compared the time of the procedure (morning vs. afternoon) using a chi-square-test and the length of the surgery with an independent sample t-test.

To compare the subjective course of anxiety on the day of the caesarean, two 2 × 3 mixed-factorial ANOVAs with the between-subject factor *group* (music group vs control group) and the within-subject factor *measurement time point* (admission, skin suture, 2 h post-surgery) and the dependent variables STAI-state and VAS-A were calculated. If sphericity was not met, a correction of the degrees of freedom according to Greenhouse-Geisser was carried out. For the objective variables heart rate and systolic and diastolic blood pressure, 2 × 2 mixed-factorial ANOVAs were used with the factors *group* and *measurements time point* (skin incision and skin suture). In addition, direct group comparisons were performed with independent-samples t-tests. Amylase values were logarithmized analogously to the generally accepted approach [24].

An a-priori power analyses for sample-size estimation was calculated using G*Power (HHU, Düsseldorf, Germany) [25]. Given an expected small to medium effect size (d = 0.35), a power of 85% and an alpha-error of .05 the required (to be analysed) sample-size is 296 (148 per group).

## Results

**Fig. 2:**
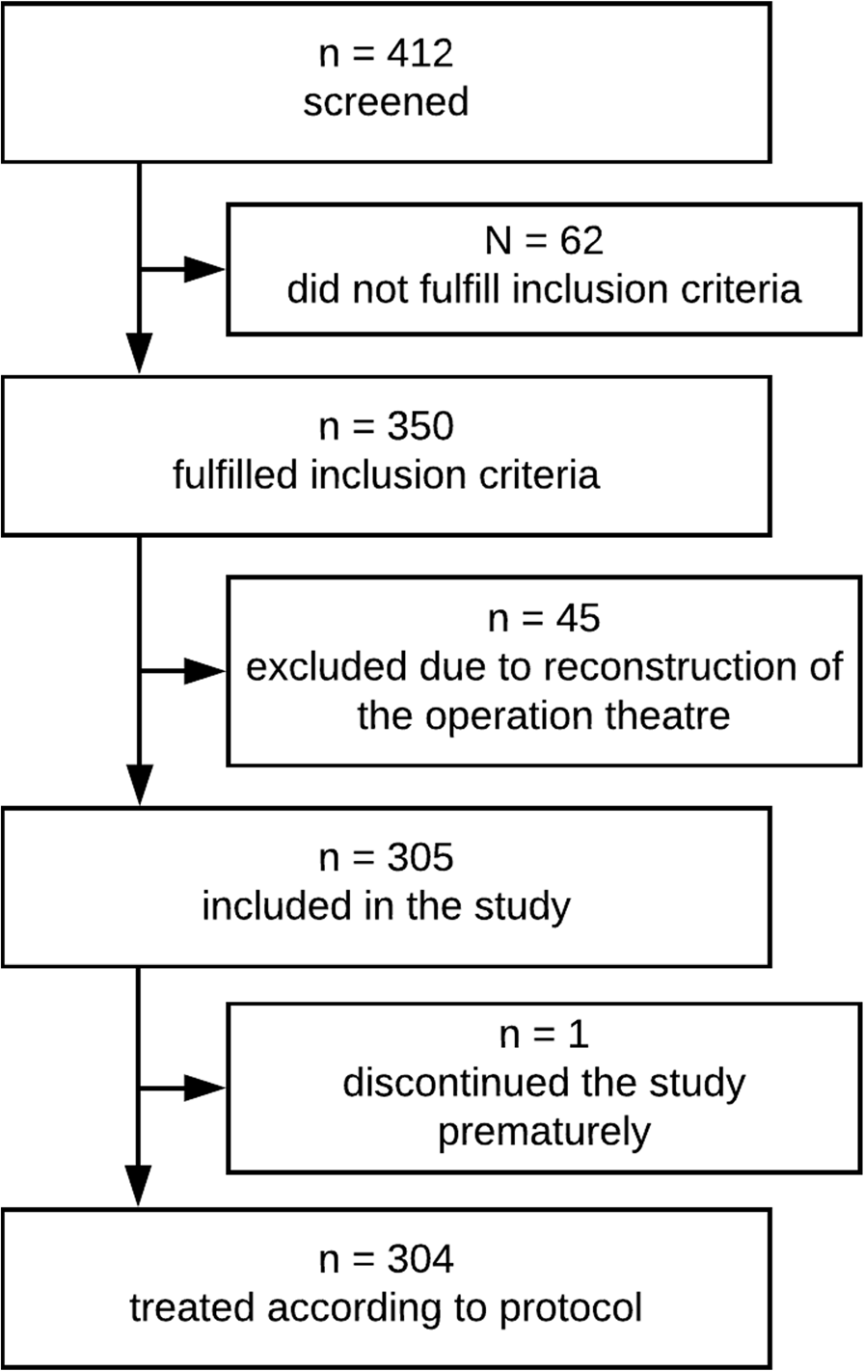
Participant flow chart

We screened and informed 412 patients about study participation. Sixty-two patients had to be excluded from further participation because they no longer fulfilled the inclusion criteria at the time of intervention (three had delivered spontaneously, 18 had an indication for caesarean preterm, 41 had a secondary caesarean (for example because of premature rupture of membranes)). Forty-five women did not take part due to technical difficulties (reconstruction of the operation theatre). One patient discontinued the study prematurely. In total 304 participants completed the study in accordance with the protocol (Fig. 2). The patients had a mean age of 33.6 years (range: 18–47 years) and a mean gestation age of 268.5 days. The data revealed that the two groups did not differ regarding the time of the procedure (morning vs. afternoon) [*χ*^*2*^(1, 301) = 0.75, *p* = .388] nor on the duration of the procedure [*t*(290) = 1.36, *p* = .175].

An overview of the descriptive data of the music and control group and the main results is given in Table 1.

**Table 1:** Overview of characteristics and results

	Music group	Control group	*P* value
Number	154	150	
Age [years]	33.5 ± 5.4 (18–47)	33.7 ± 5.4 (21–44)	.883
Gestational Age [days]	269	268	.231
Time of procedure [morning/evening]	81/73	70/77 (3 N/A)	.388
Duration of procedure [mins]	43.0	41.6	.175
STAI-Trait	36.39 ± 8.45	37.14 ± 8.74	.474
STAI-State I	47.54 ± 10.40	48.28 ± 11.79	.593
STAI-State II	31.56 ± 6.30	34.41 ± 9.23	**.004**
STAI-State III	29.54 ± 5.91	30.91 ± 7.14	.140
VAS-A I [cm]	4.83 ± 2.61	5.18 ± 2.89	.349
VAS-A II [cm]	1.27 ± 1.20	1.76 ± 1.78	**.018**
VAS-A III [cm]	0.69 ± 0.88	1.04 ± 1.29	**.018**
C-increase I to II [nmol/L]	12.29 ± 12.15	16.61 ± 16.14	**.043**
C-decrease II to III [nmol/L]	− 13.77 ± 13.05	−17.07 ± 16.80	.128
sAA-increase [ln] I to II	1.58 ± 1.40	1.76 ± 1.24	.414
sAA-decrease II to III	−0.31 ± 1.27	−0.57 ± 1,10	.165
RR sys skin incision [mmHg]	130.11 ± 14.97	136.19 ± 16.57	**.002**
RR sys II [mmHg]	121.42 ± 12.89	121.58 ± 13.00	.981
RR dia skin incision [mmHg]	70.82 ± 9.35	72.77 ± 10.55	.106
RR dia II [mmHg]	64.65 ± 9.61	64.99 ± 9.21	.764
HR skin incision [1/min]	88.40 ± 16.23	92.57 ± 18.26	**.049**
HR II [1/min]	77.97 ± 13.91	80.51 ± 14.23	.141

N/A: data not available; I: at admission; II: at skin suture; III: 2 h post-surgery; VAS-A: Visual Analogue Scale for Anxiety; C: salivary cortisol; sAA: salivary alpha amylase; RR sys: systolic blood pressure; RR dia: diastolic blood pressure; HR: heart rate

Note: bold values indicate a significant difference between groups

### Subjective parameters

**Fig. 3:**
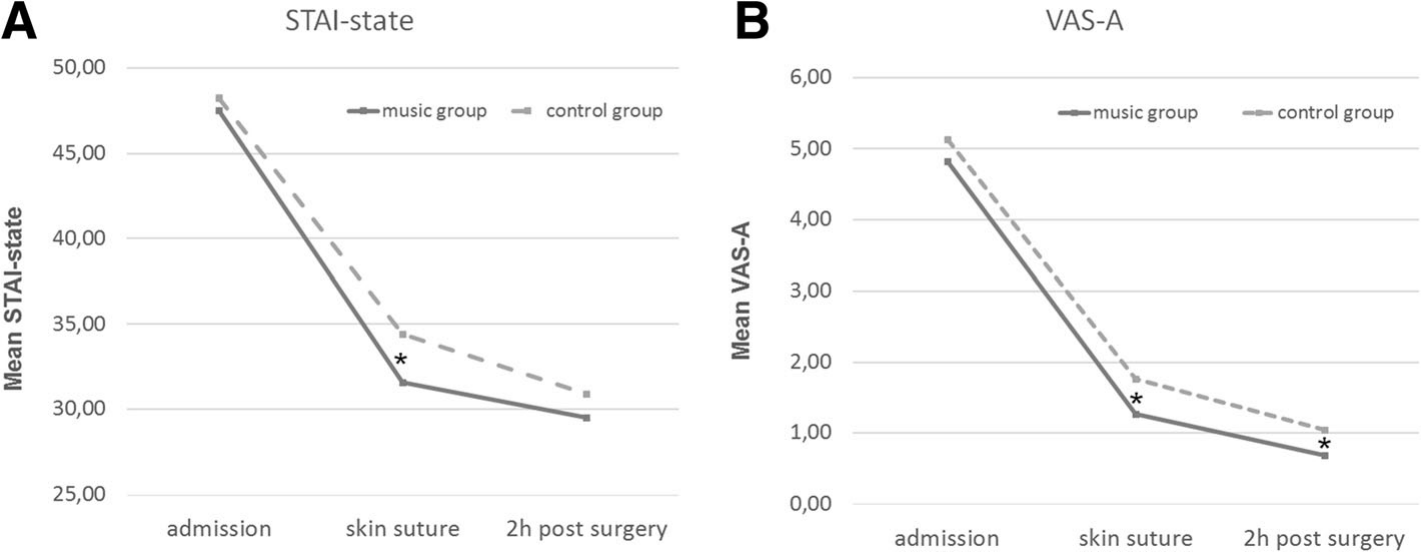
The subjective course of anxiety for both groups. **a** For the STAI-state scores the music group displayed significantly lower anxiety levels at skin suture than the control group. **b** For the VAS-A scores anxiety levels of the music group are significantly below the control group at skin suture and remain lower two hours after the caesarean. * *p* < .05

For the STAI-state, significant main effects were found for the factors *measurement time point* [*F* (1.78, 451.28) = 454.35, *p* < .001] and *group* [*F* (1.254) = 4.12, *p* = .043]. The interaction was not significant [*F* (1.78, 451.28) = 1.47, *p* = .230]. Post-hoc comparisons showed that the two groups did not differ on admission (*p* = .593). At skin suture the music group showed significantly less anxiety compared to the control group [*t* (254) = 2.88, *p* = .004, mean difference = 2.72, 95% CI (0.76, 4.67), d = 0.36]. 2 h after surgery there was no significant difference *(p* = .140) (Fig. 3a).

For the VAS-A, the factors *measurement time point* [*F* (1.39, 301.32) = 378.50, *p* < .001] and *group* [*F* (1.217) = 4.51, *p* = .035] showed significant effects, whereas no interaction was present [*F* (1.39, 301.32) = 0.17, *p* = .847]. A post-hoc t-test showed that the level of anxiety in the two groups did not differ at admission (*p* = .349). At skin suture [*t* (217) = 2.39, *p* = .018, mean difference = 0.49, 95% CI (0.08, 0.89), d = 0.32] and 2 h post-surgery [*t* (217) = 2.38, *p* = .018, mean difference = 0.35, 95% CI (0.06, 0.64), d = 0.32] there were significant differences in favour of the music group (Fig. 3b).

### Objective parameters

**Fig. 4:**
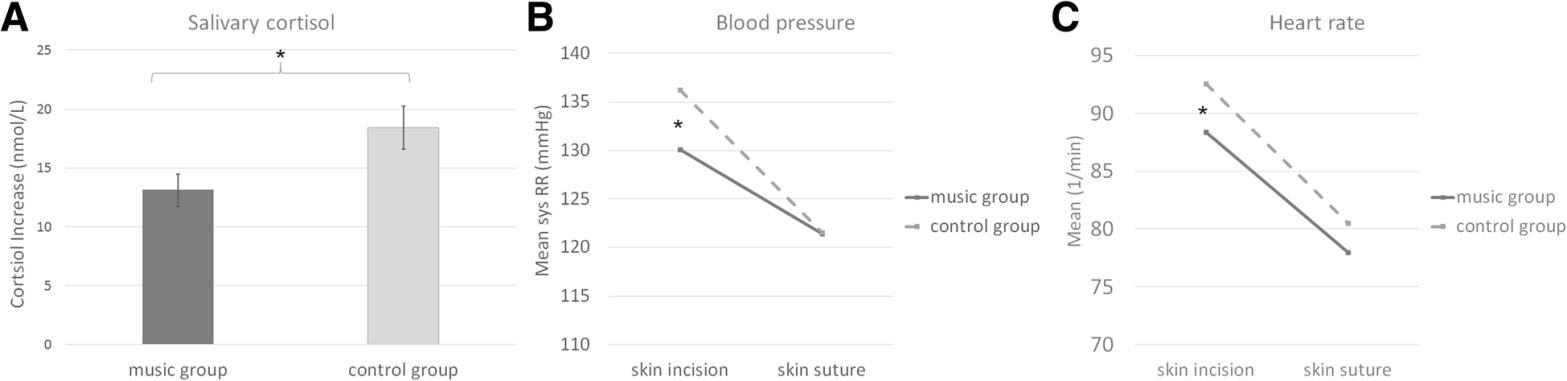
Objective Measures. **a** The increase of salivary cortisol from admission to skin suture is significantly higher in the control group than the music group. **b** The music group show significantly lower systolic blood pressure at skin incision. **c** Heart rate is significantly lower in the music group than the control group at skin incision

Cortisol increase from admission to skin suture was significantly lower in the music group than in the control group [*t* (181) = 2.04, *p* = .043, mean difference = 4.32, 95% CI (0.14, 8.49), d = 0.30] (Fig. 4a). The decrease in cortisol from skin suture to 2 h post-surgery did not differ significantly between the two groups [t (193) = 1.53, *p* = .121, mean difference = 3.30, 95% CI (− 0.95, 7.55), d = 0.22]. The increase in amylase from admission to skin suture and decrease to 2 h after surgery did not differ between the two groups (*p*-values > .165).

For systolic blood pressure, significant main effects were found for *group* [*F* (1,270) = 4.73, *p* = .030] and *measurement time point* [*F* (1,270) = 132.58, *p* < .001] and a significant interaction revealed [*F* (1,270) = 8.57, *p* = .004]. At skin incision the mean systolic blood pressure was significantly lower in the music group (M = 130.11 mmHg) than in the control group (M = 136.19 mmHg) [*t* (270) = 3.18, *p* = .002, mean difference = 6.35, 95% CI (2.59, 10.11), d = 0.39] (Fig. 4b). There was no difference at skin suture (*p* = .981). For the diastolic blood pressure value, no significant group differences were found (p-values > .197).

Also for heart rate significant effects of the factors *group* [*F* (1,266) = 4.51, *p* = .035] and *measurement time point* [*F* (1,266) = 104.99, *p* < .001] were revealed. There was no significant interaction [*F* (1,266) = 0.55, *p* = .457]. Post-hoc comparisons showed that at skin incision the heart rate was significantly lower in the music group (M = 88.40) than in the control group (M = 92.57) [t (269) = 1.98 *p* = .049, mean difference = 4.13, 95% CI (0.02, 8.24), d = 0.24]. At skin suture, the groups did not differ significantly (*p* = .141) (Fig. 4c).

### Acceptance of music intervention

Of the participants in the music group 95.5% stated that they would listen to music again at a possible next caesarean. Eighty-six percent were satisfied with the choice of music. In addition, 89.7% said that the music made the situation more enjoyable and 73.4% thought the music had calmed them.

## Discussion

Overall, the study shows, in subjective as well as objective dimensions, an anxiety and stress reducing effect of music during caesarean.

While both groups showed high baseline values for STAI-state and VAS-A without significantly differing from each other at admission, the subjective anxiety drop in both, STAI-state and VAS-A, was significantly higher in the music group and resulted in significantly lower anxiety scores compared to the control group at skin suture. The shown positive effect of music on these subjective measures for anxiety and stress is in line with previous studies investigating the effect of music in the context of caesarean and other medical procedures [11, 13, 14, 16, 23, 26]. One of these studies showed a persisting positive effect for six hours [16]. Our data support this observation insofar that women in the music group had significantly lower scores on the VAS-A for another two hours after skin suture. This effect was not found for the STAI-state. It is likely that the STAI-state is less appropriate than the VAS-A in the obstetrical environment due to the duration of the survey?

In terms of objective parameters, the music group showed a significantly lower increase in salivary cortisol than the control group from admission to skin suture. Saliva cortisol measures the impact of a stressor with a latency of about 30 min [27]. In this respect, the measurement at skin suture represents a large part of the objective stress sensation during the operation [2]. Therefore, the present result reflects that the music group also objectively experiences reduced stress levels during the caesarean. A lowering effect of music on cortisol could also be shown in studies in other medical fields [28].

A comparable effect could not be found for salivary amylase. Whereas cortisol represents the activity of the hypothalamic-pituitary-adrenocortical axis, salivary amylase represents the activation of the sympathetic nerve system [29]. In line with previous work on stress during surgical procedures, our study shows no measurable difference in salivary amylase between the groups [30, 31]. This might be an indication that there are unrecognized factors, which impede a reliable interpretation of the salivary amylase during surgical procedures.

A variety of studies have examined the influence of music on the cardiovascular system [32]. In addition to a direct impact on the dopaminergic mesolimbic reward centre [33], an interaction of the external musical rhythm with the internal body rhythms of heart and respiratory rate as a main carrier of the effect of music is debated [34–36]. Thereby, the value of systolic blood pressure and heart rate is well documented as objective parameters reflecting stress and anxiety [32]. In the present study, the music group showed significantly lower heart rate and systolic blood pressure levels at skin incision. This took place on average 18 min after the patient had been admitted to the operating room and the start of the music intervention. Therefore, we would argue that the calming effect of music on the expectant mother was already present at skin incision. In the case of skin suture, however, no significant difference was detectable. At this time, the new-born was usually already in the arms of the mother, so that pulse and blood pressure are certainly subject to an undetermined bias.

Besides the positive influence of music on the measured stress and anxiety of patients, the high acceptance of the intervention should also be noted. Ninety-six percent of women in the intervention group would want to hear music again during a possible repeated caesarean, regardless of any therapeutic effects. The positive response of the women regarding the music intervention alone are gratifying and should encourage the use of music in obstetrics and further research in this area.

A limitation of the study is the lack of opportunity to gain an even more comprehensive picture of the course of anxiety during caesarean through measuring more time points. For example a measurement of anxiety levels just before the women enters the operating theatre would be desirable as this time point may be the most anxious time for the women. This is partially compensated by the fact that the different measuring instruments used in this study have different latencies and thus cover a large period of time around the intervention. Additionally, in order to ensure a trial close to normal conditions of a caesarean and the needs of the woman, we decided not to use headphones, in contrast to other studies [18]. Naturally, this impeded blinding of the study staff. Using group blinded analysis and application of objective parameters such as saliva cortisol, this disadvantage was accounted for as far as possible. Another limitation which could have influenced the study results is the lack of data on the relationship and role of the nurses and other health professionals in usual interaction and information sharing with the woman during and after procedure.

We would like to emphasize that with a large sample of 304 participants and several measurement time points throughout the day of the caesarean as a strength of the study. This is the first study in this area of research that has the necessary power to detect even small effect sizes.

## Conclusions

In view of the results, the present study should have implications for clinical practice. Music is a simple, inexpensive, effective and safe intervention that can be easily implemented in everyday clinical practice. Therefore, it would be desirable if the possibility to listen to music could be routinely offered to women giving birth by caesarean. However, further research should consider the impact of music on the surgical team. Although previous work showed adverse effects only for high volumes and complex interventions [37], this has not been investigated explicitly in the context of caesarean.

In conclusion, the results of the present study show that music during caesarean has an anxiety and stress soothing effect on the wake patient. Implementation in clinical routine therefore seems advisable.

## Abbreviations

ANOVA: Analysis of variance
C: Salivary cortisol
CONSORT: Consolidated Standards of Reporting Trials
HR: Heart rate
Pts: Patients
RR dia: Diastolic blood pressure
RR sys: Systolic blood pressure
sAA: Salivary alpha amylase
SD: Standard deviation
STAI: State-Trait Anxiety Inventory
VAS-A: Visual analogue scale for anxiety
